# Supplementary figures and images for: Cell cycle length governs heterochromatin reprogramming during early development in non-mammalian vertebrates
Source: EMBO Rep. 2024 Jun 28;25(8):11. doi: 10.1038/s44319-024-00188-5 (PMC11315934; doi:10.1038/s44319-024-00188-5)

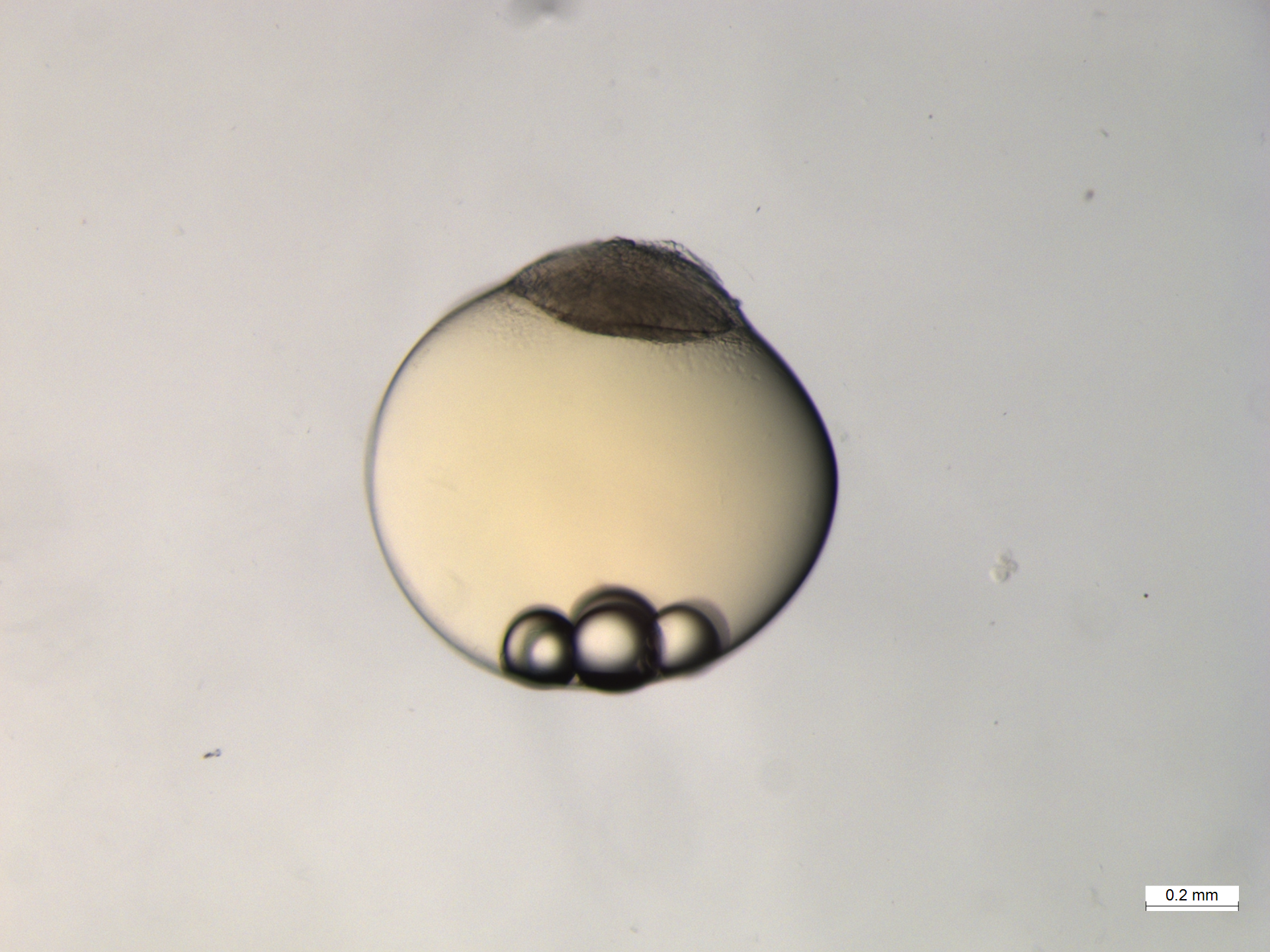

Supplement: Supplementary file 5 — Source data Fig. 2 [file 44319_2024_188_MOESM5_ESM.zip › Figure 2/2B/Ama_12.5hpf.tif]

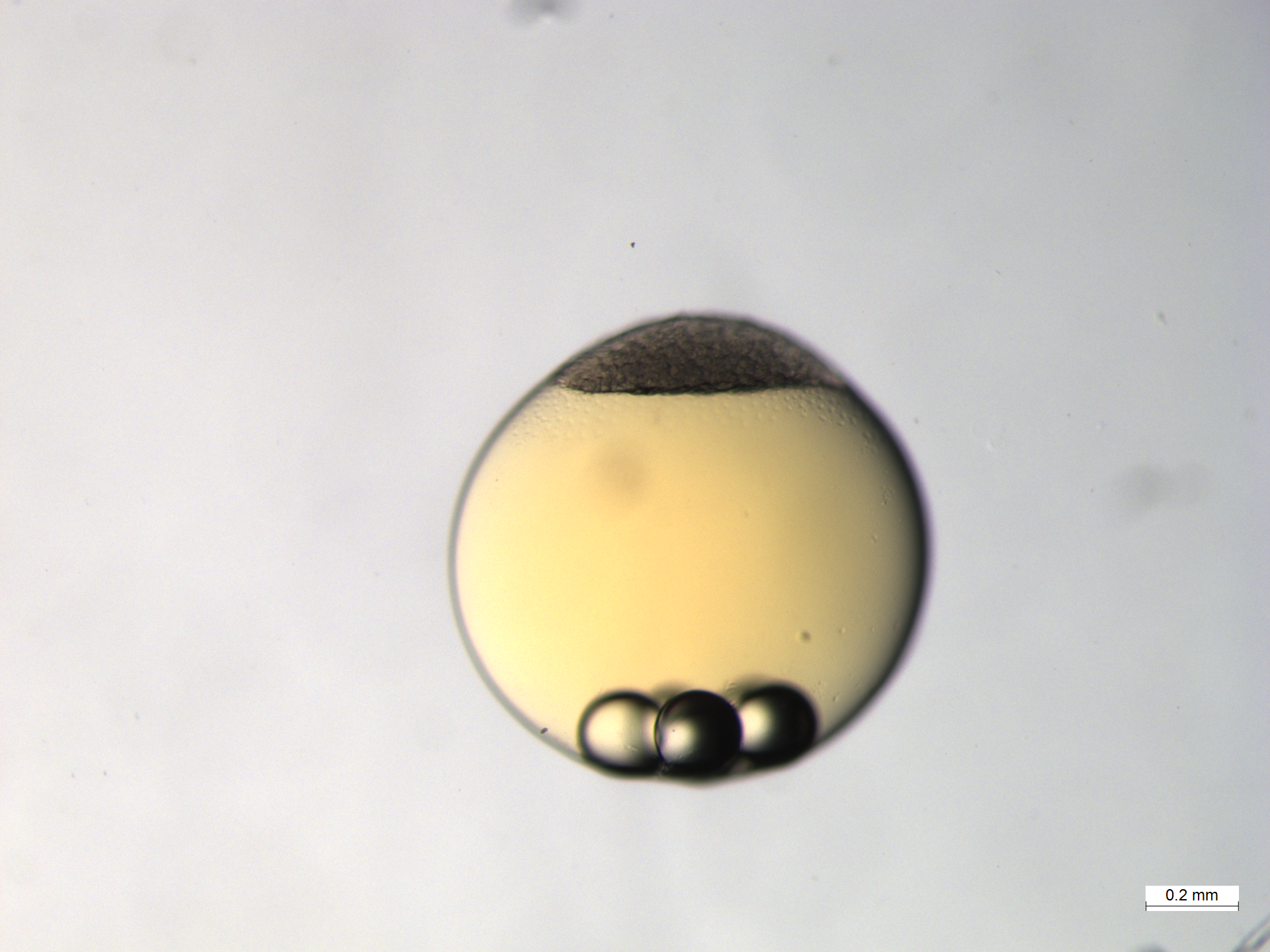

Supplement: Supplementary file 5 — Source data Fig. 2 [file 44319_2024_188_MOESM5_ESM.zip › Figure 2/2B/Control_8.5hpf.tif]

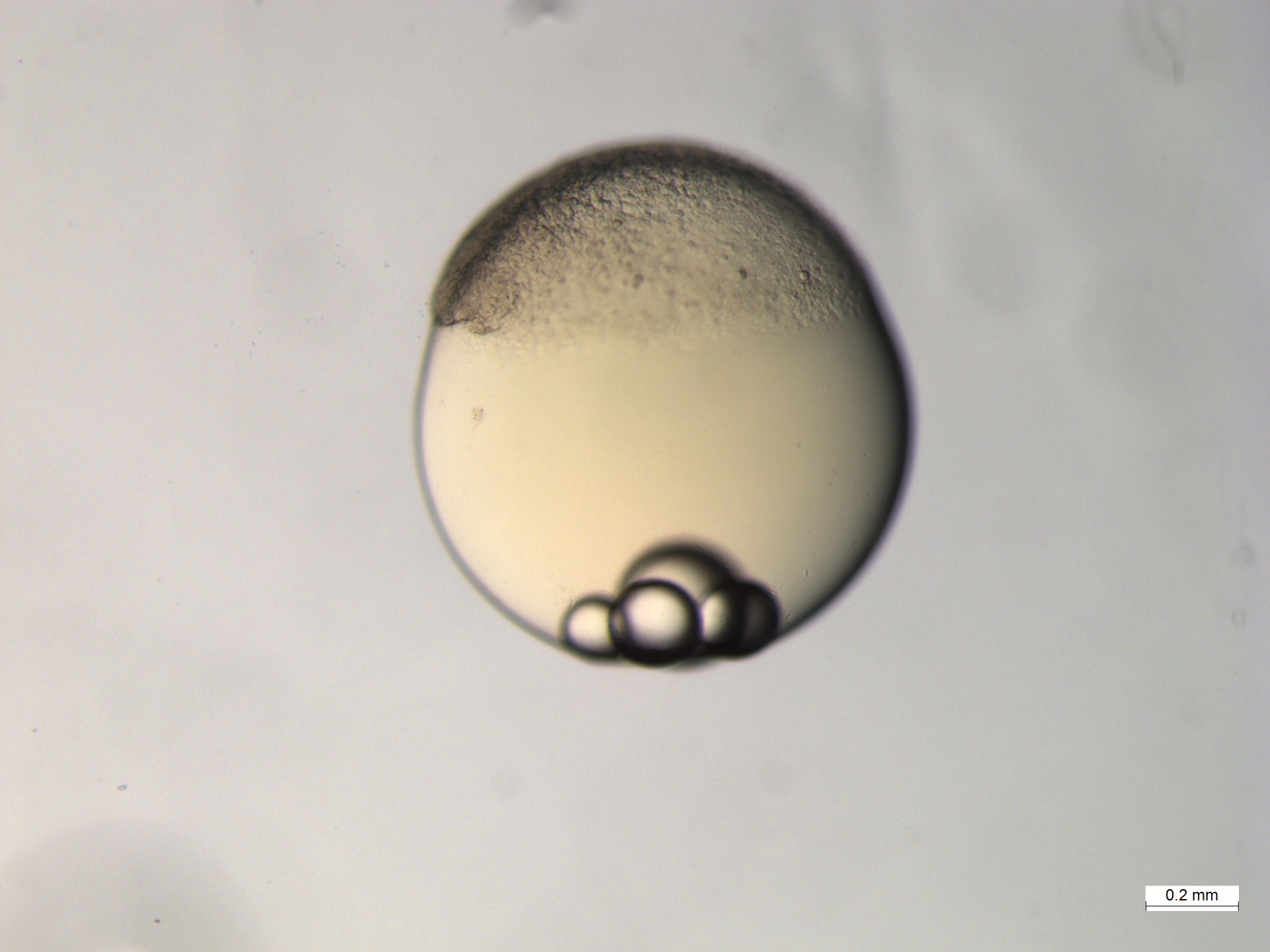

Supplement: Supplementary file 5 — Source data Fig. 2 [file 44319_2024_188_MOESM5_ESM.zip › Figure 2/2B/Control_12.5hpf.tif]

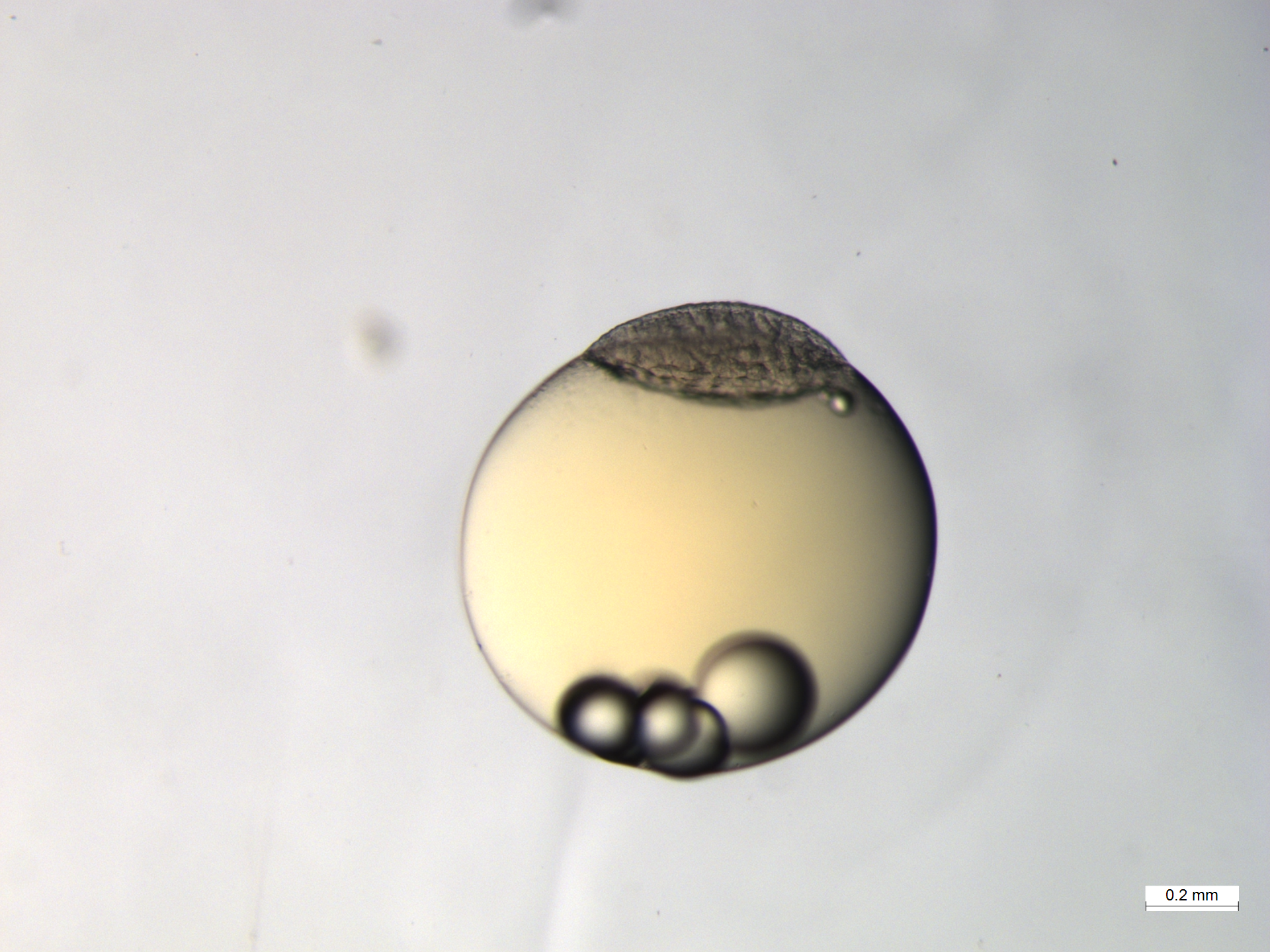

Supplement: Supplementary file 5 — Source data Fig. 2 [file 44319_2024_188_MOESM5_ESM.zip › Figure 2/2B/Ama_8.5hpf.tif]

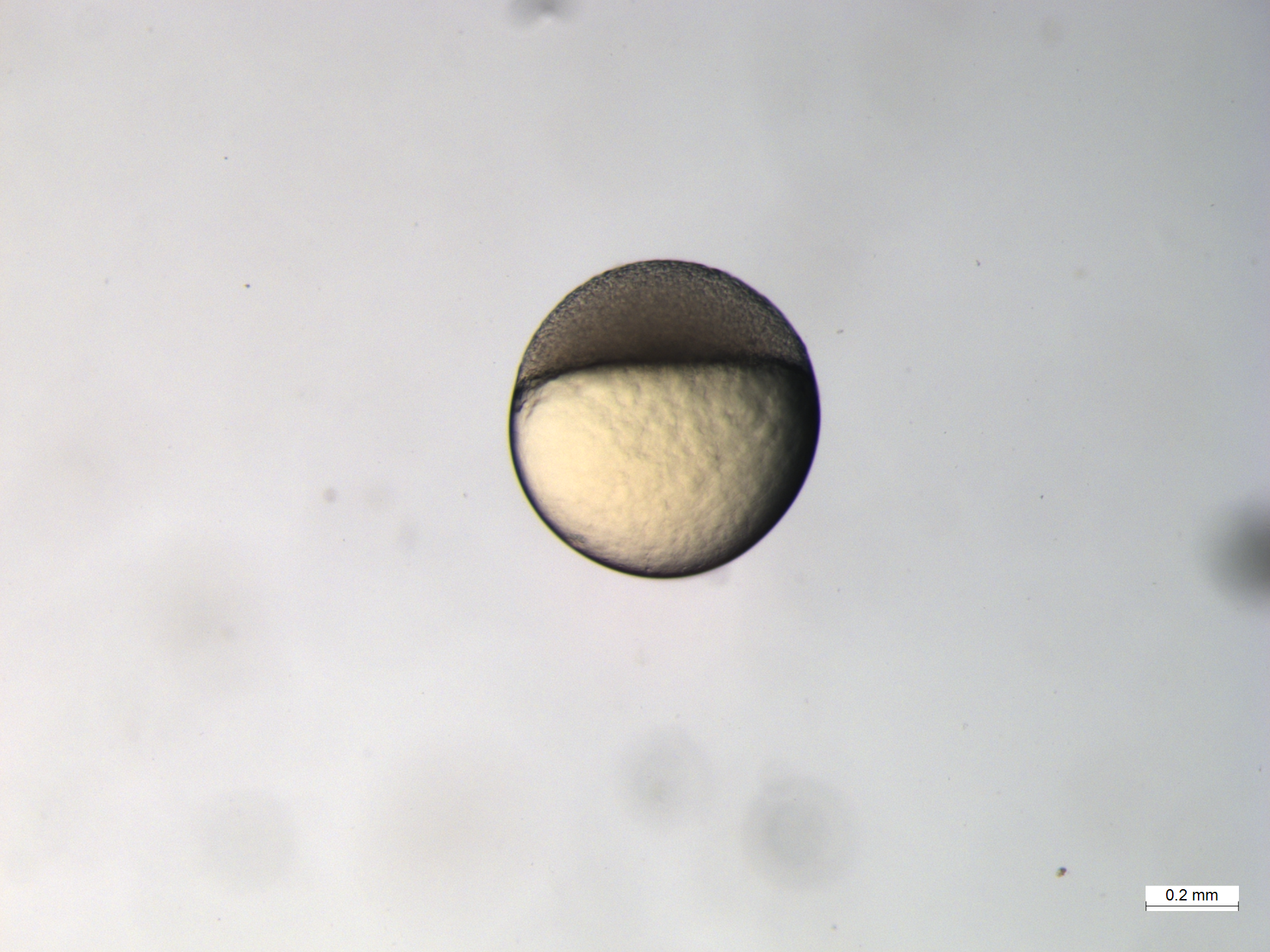

Supplement: Supplementary file 8 — Source data Fig. 5 [file 44319_2024_188_MOESM8_ESM.zip › Figure 5/5D/Control_4hpf.tif]

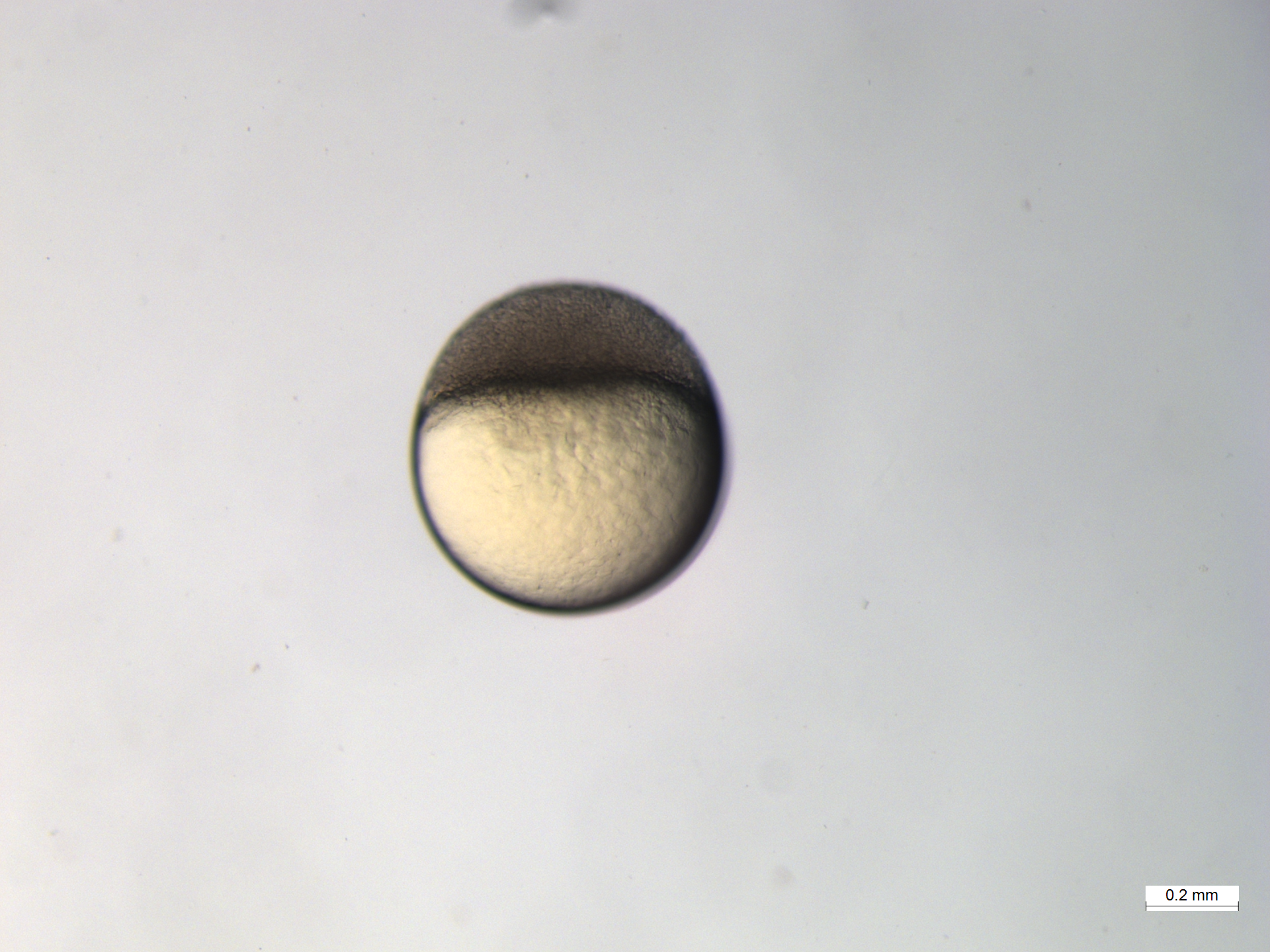

Supplement: Supplementary file 8 — Source data Fig. 5 [file 44319_2024_188_MOESM8_ESM.zip › Figure 5/5D/Ama_5.2hpf.tif]

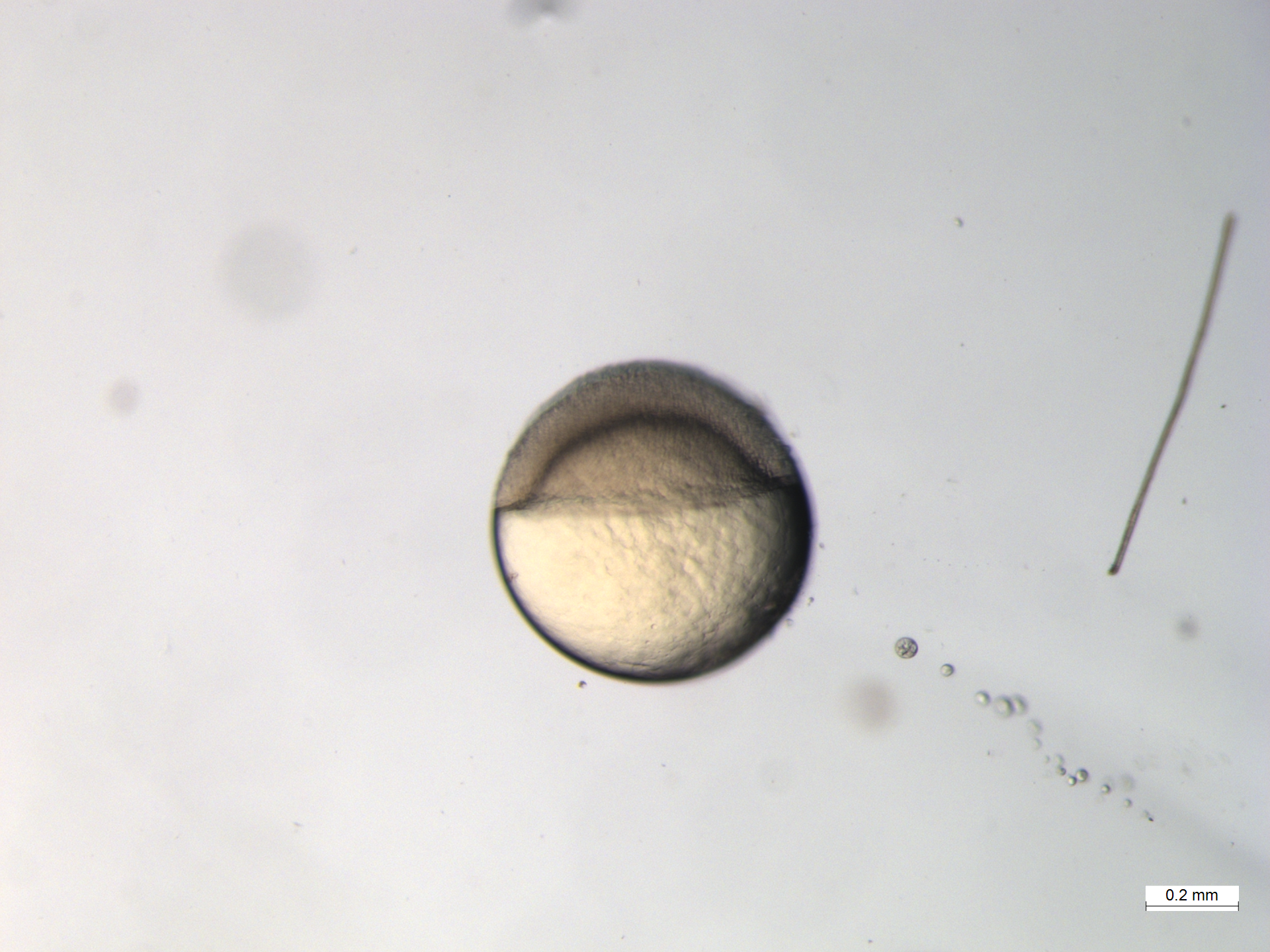

Supplement: Supplementary file 8 — Source data Fig. 5 [file 44319_2024_188_MOESM8_ESM.zip › Figure 5/5D/Control_5.2hpf.tif]

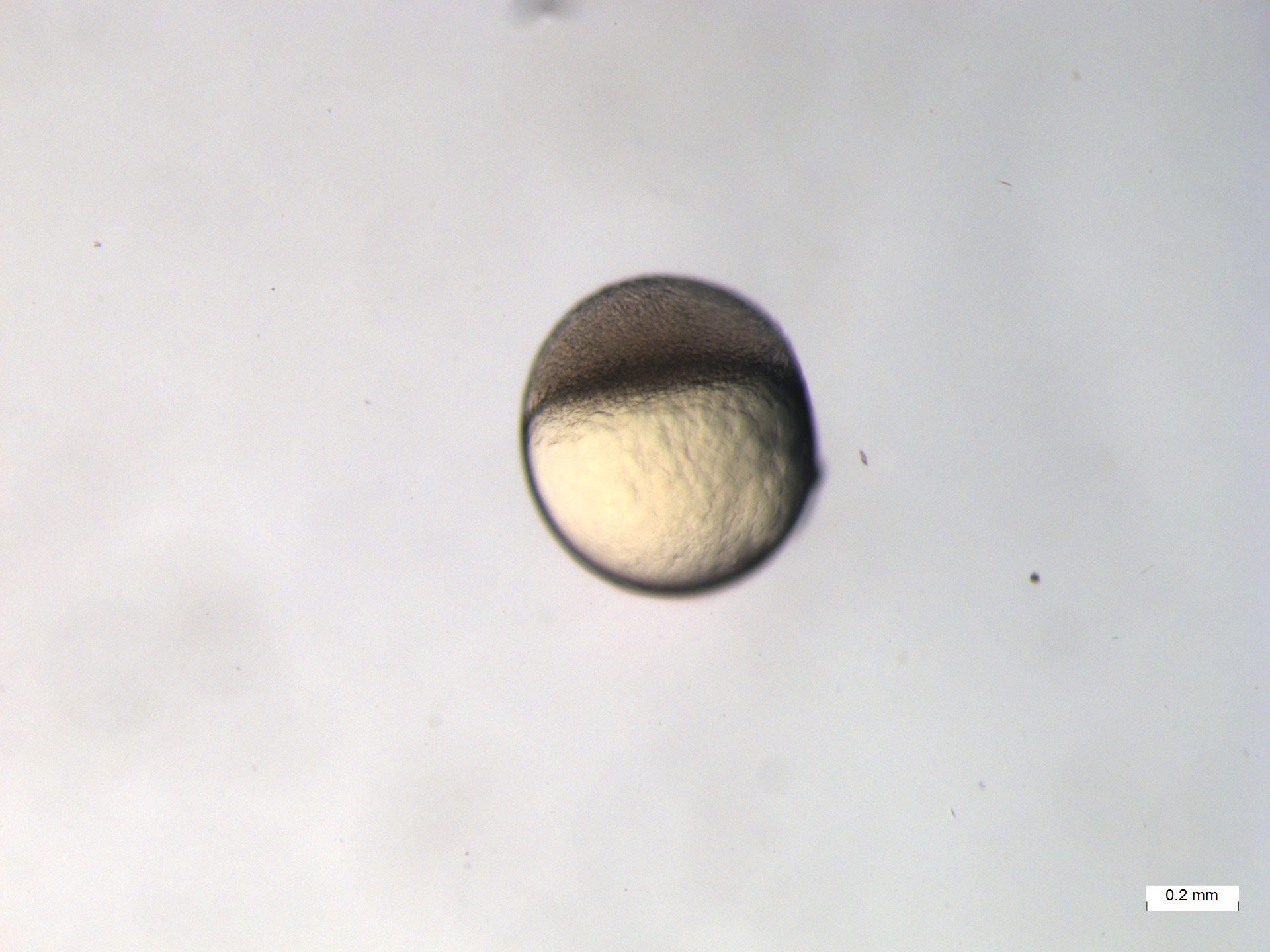

Supplement: Supplementary file 8 — Source data Fig. 5 [file 44319_2024_188_MOESM8_ESM.zip › Figure 5/5D/Ama_4hpf.tif]

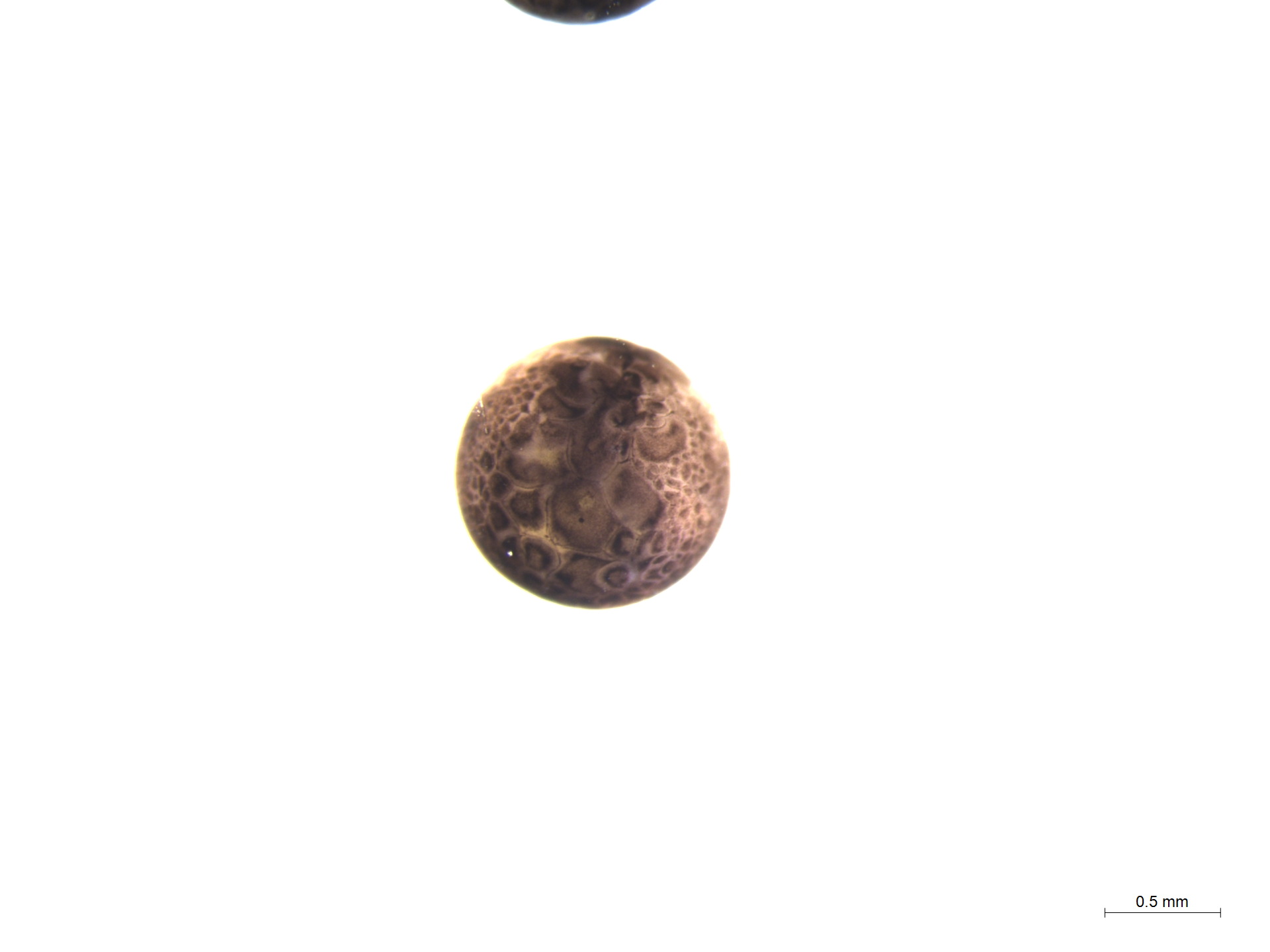

Supplement: Supplementary file 9 — Source data Fig. 6 [file 44319_2024_188_MOESM9_ESM.zip › Figure 6/6E/Chk1_9hpf.tif]

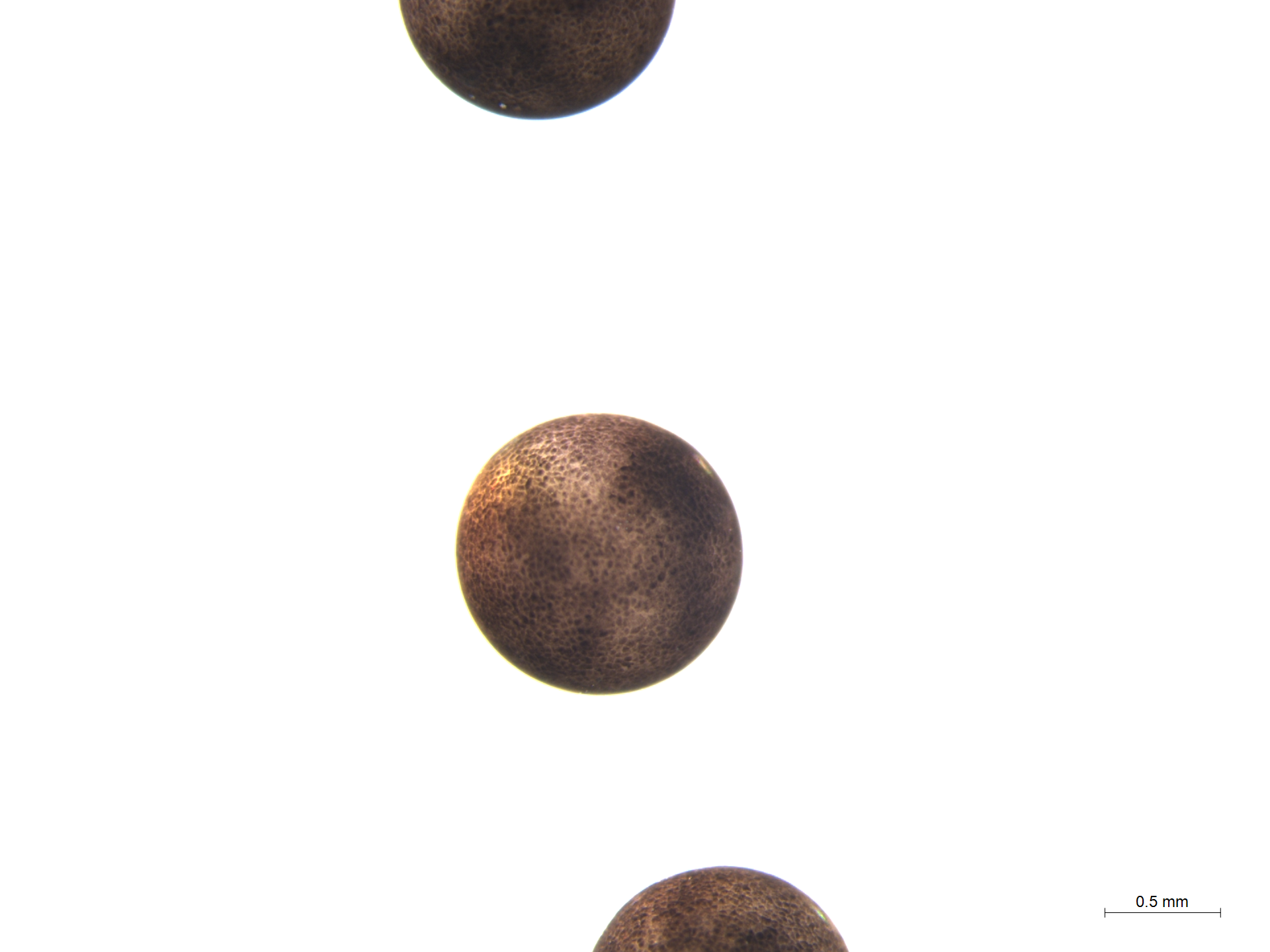

Supplement: Supplementary file 9 — Source data Fig. 6 [file 44319_2024_188_MOESM9_ESM.zip › Figure 6/6E/Control_9hpf.tif]

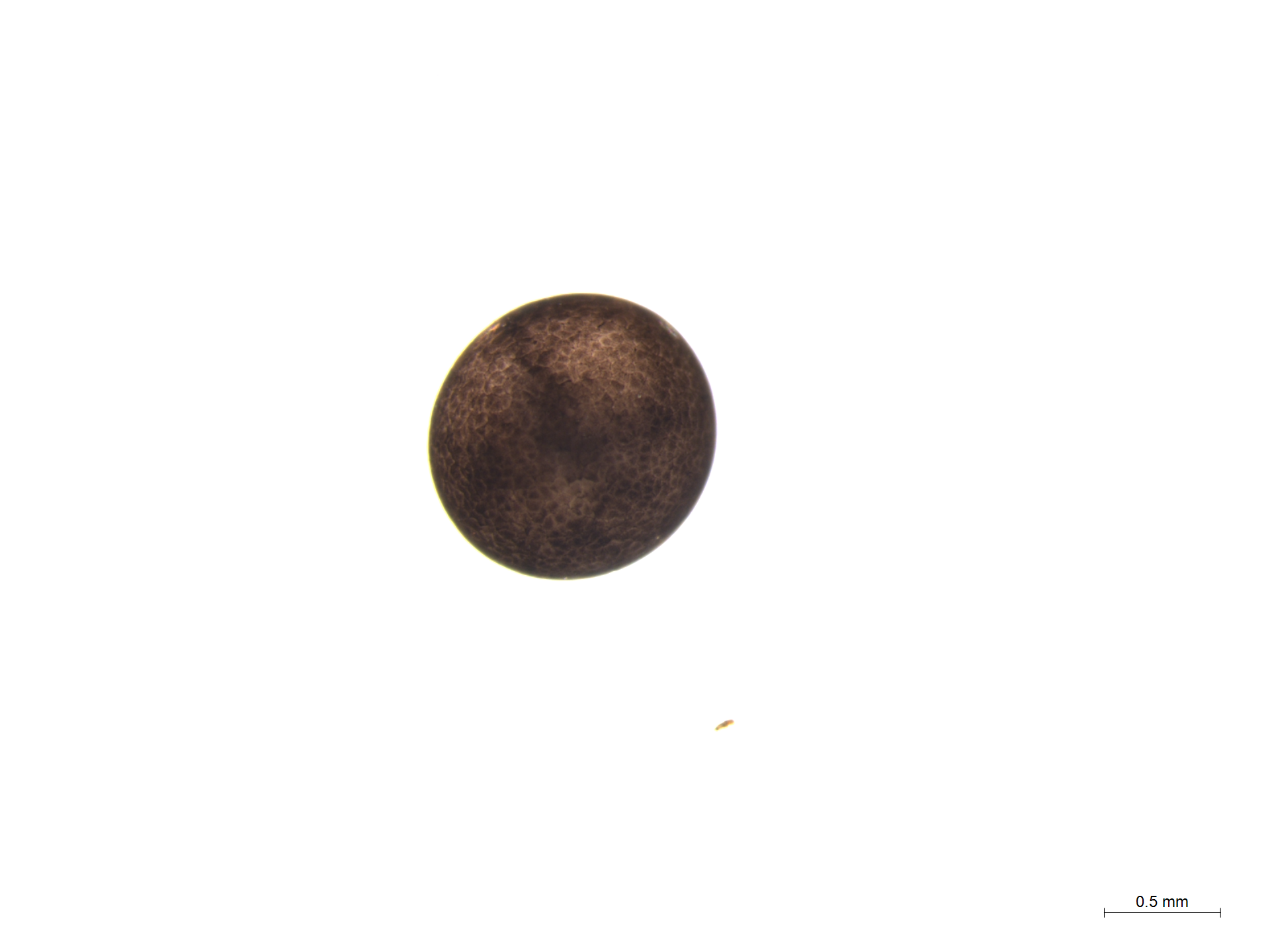

Supplement: Supplementary file 9 — Source data Fig. 6 [file 44319_2024_188_MOESM9_ESM.zip › Figure 6/6E/Control_7hpf.tif]

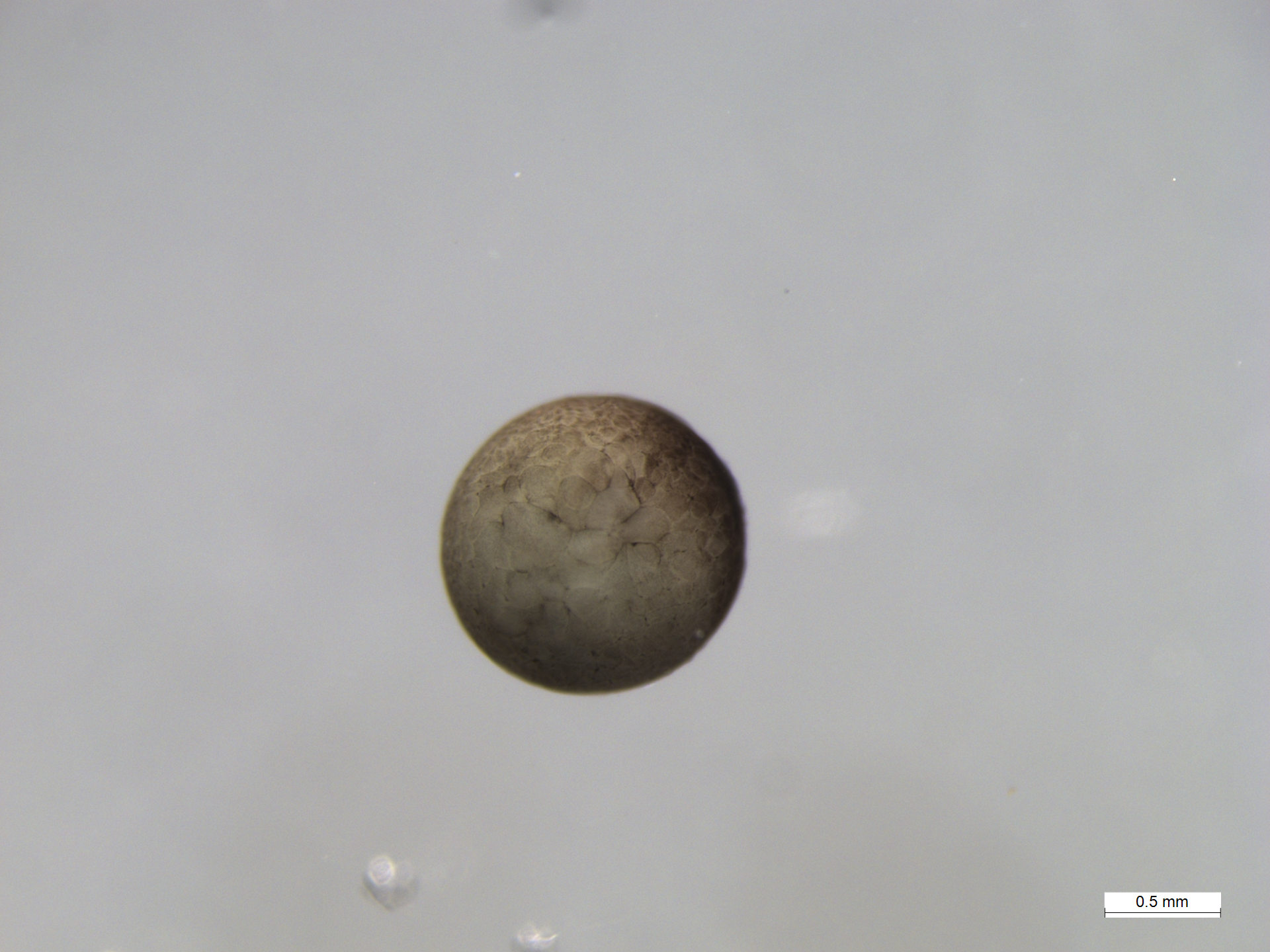

Supplement: Supplementary file 9 — Source data Fig. 6 [file 44319_2024_188_MOESM9_ESM.zip › Figure 6/6D/Ama_7hpf.tif]

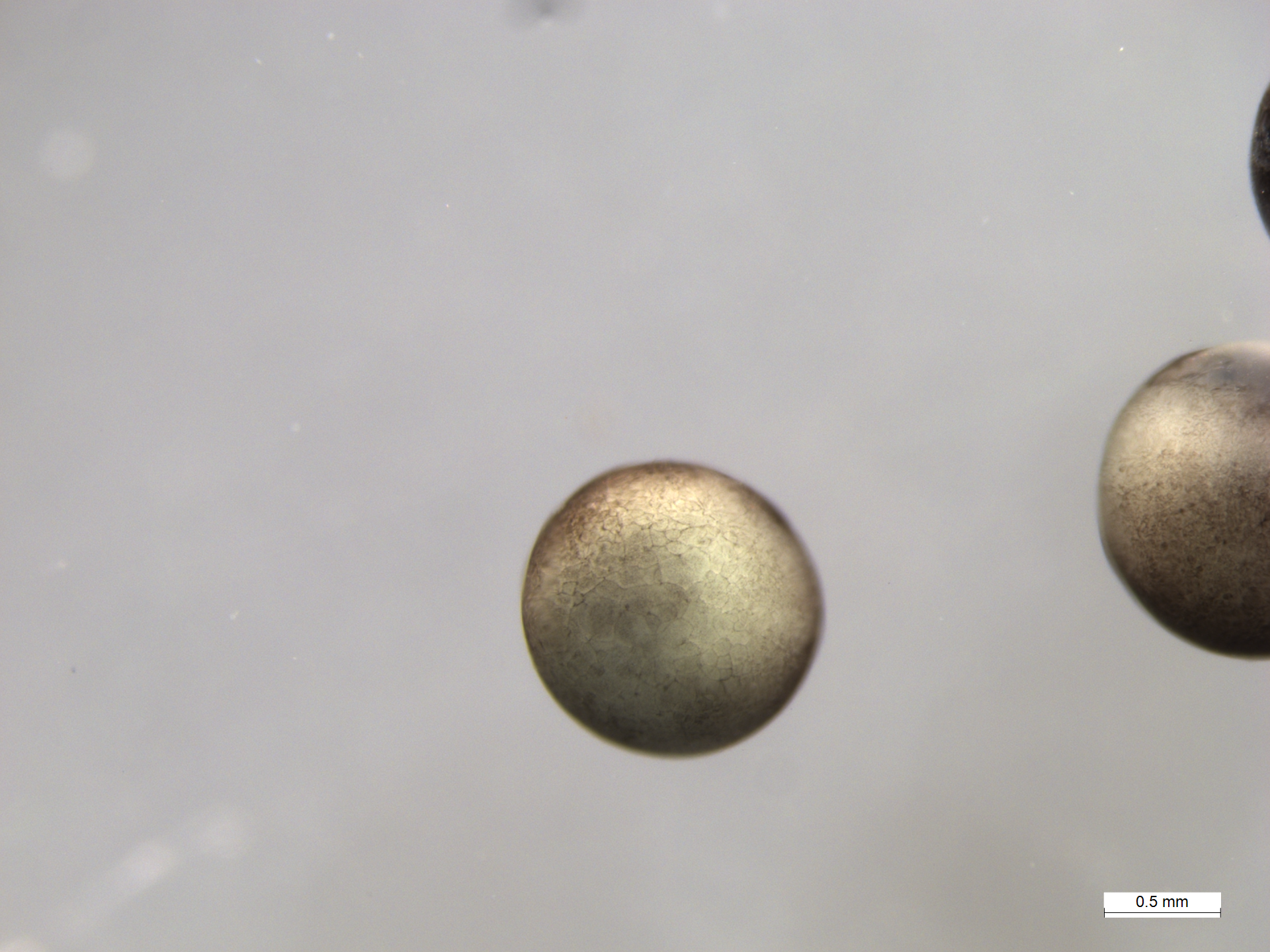

Supplement: Supplementary file 9 — Source data Fig. 6 [file 44319_2024_188_MOESM9_ESM.zip › Figure 6/6D/Ama_10.5hpf.tif]

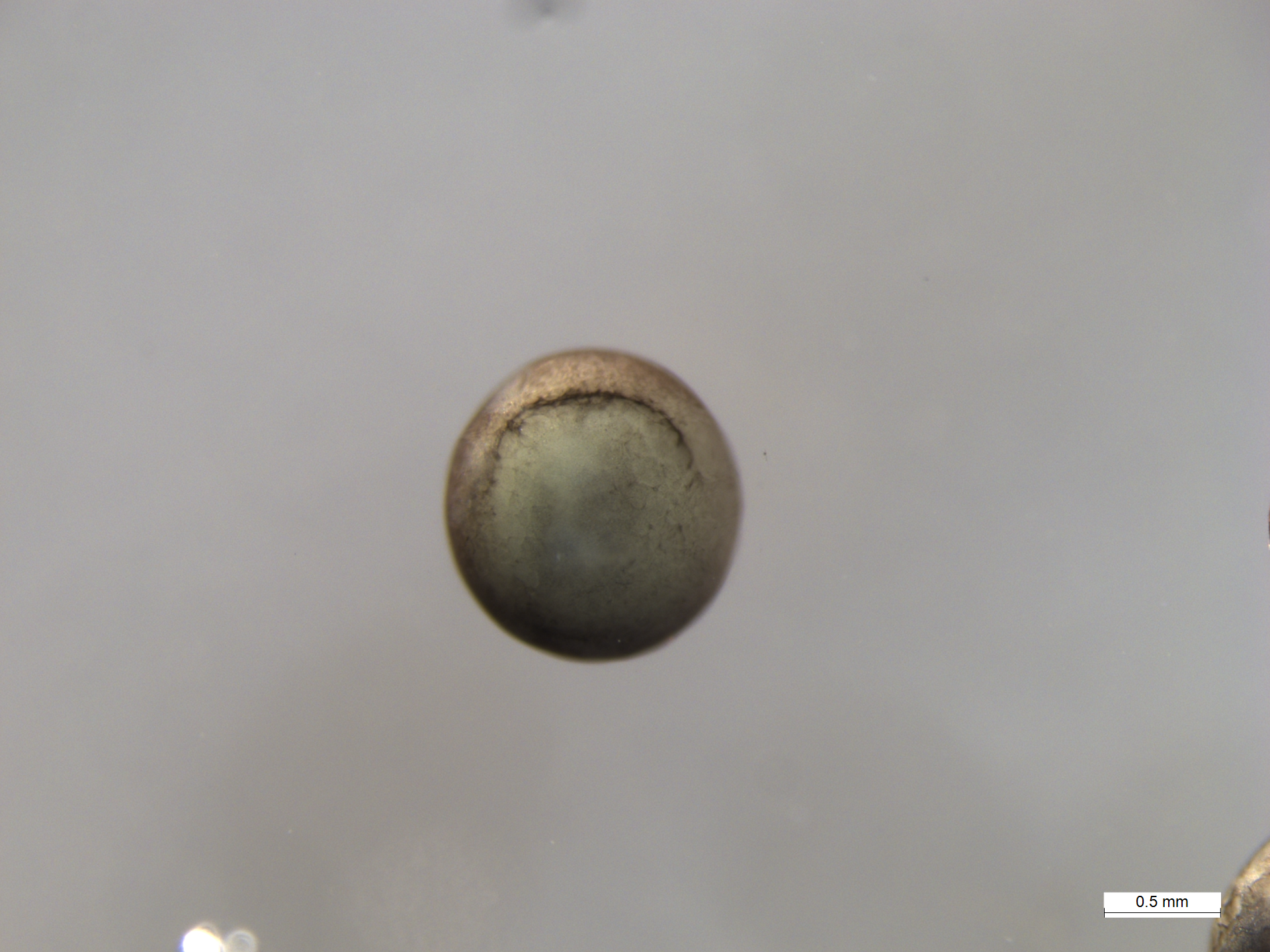

Supplement: Supplementary file 9 — Source data Fig. 6 [file 44319_2024_188_MOESM9_ESM.zip › Figure 6/6D/Control_10.5hpf.tif]

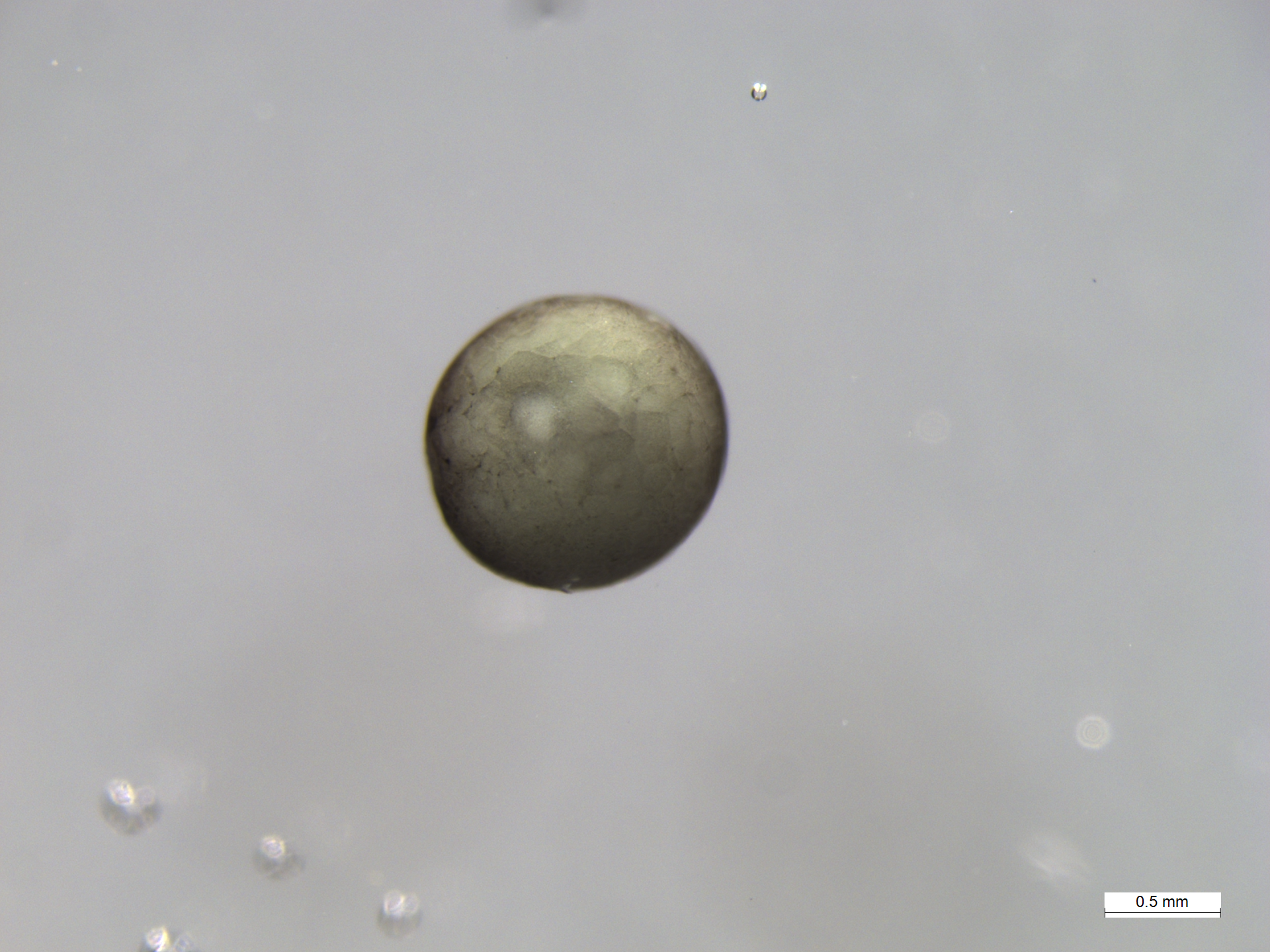

Supplement: Supplementary file 9 — Source data Fig. 6 [file 44319_2024_188_MOESM9_ESM.zip › Figure 6/6D/Control_7hpf.tif]
