# Supplementary material for: Cell cycle length governs heterochromatin reprogramming during early development in non-mammalian vertebrates
Source: EMBO Rep. 2024 Jun 28;25(8):11. doi: 10.1038/s44319-024-00188-5 (PMC11315934; doi:10.1038/s44319-024-00188-5)
Supplement: Supplementary file 10 — Expanded View Figures [file 44319_2024_188_MOESM10_ESM.pdf]

Expanded View Figures

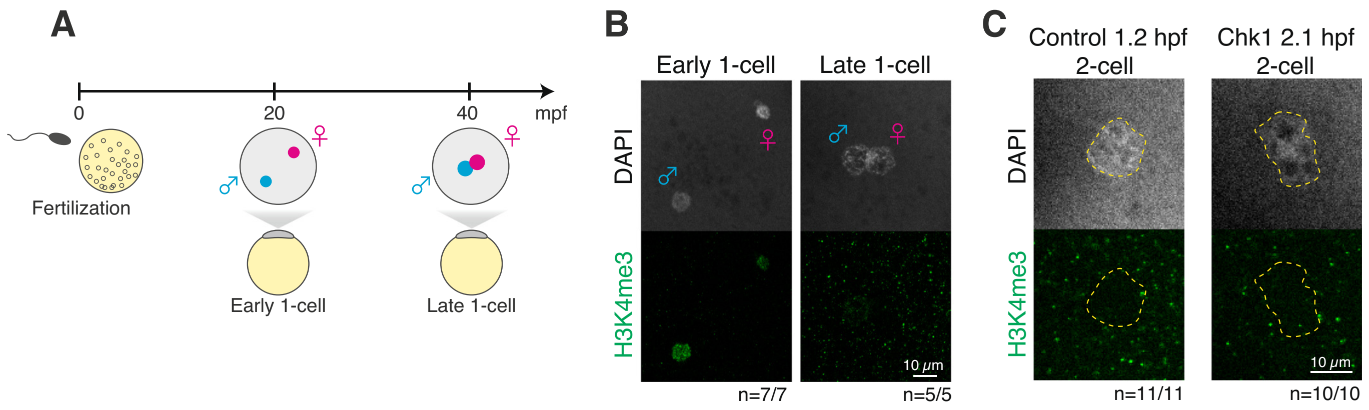

Figure EV1. Supportive data for Fig. 1.

(**A**) Development of medaka embryos at the one-cell stage. Blue and magenta indicate paternal and maternal pronuclei, respectively. mpf: minutes post fertilization. (**B**) Immunofluorescence staining of H3K4me3 at the one-cell stage. The number of embryos with the representative pattern is indicated at the bottom. (**C**) Immunofluorescence staining of H3K4me3 at the 2-cell stage in the chk1 experiment. Yellow dashed line indicates nuclei. The number of embryos with the representative pattern is indicated at the bottom.

© The Author(s)

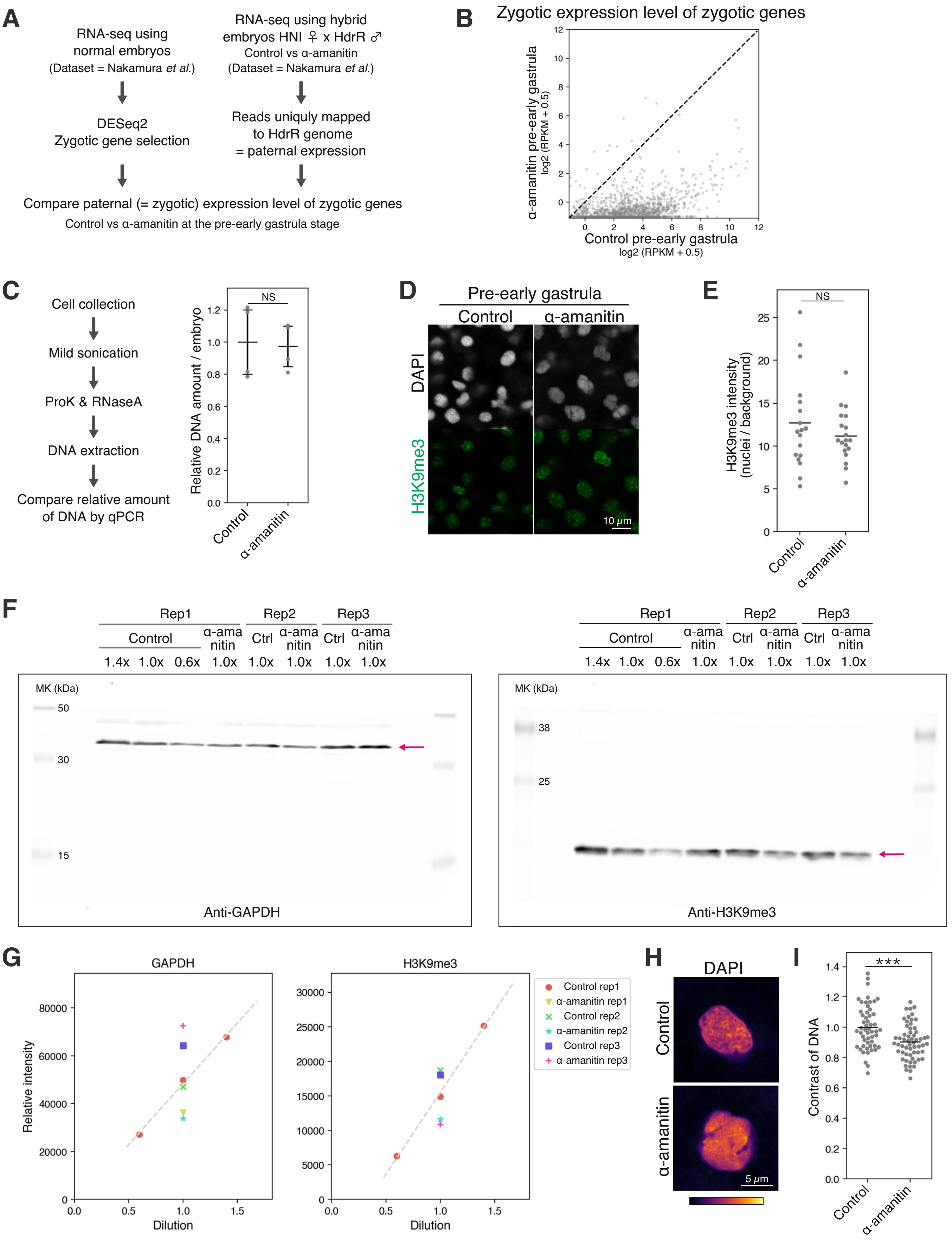
A
RNA-seq using
normal embryos
(Dataset = Nakamura et al.)
DESeq2
Zygotic gene selection
RNA-seq using hybrid
embryos HNI ♀ x HdrR ♂
Control vs α-amanitin
(Dataset = Nakamura et al.)
Reads uniqluy mapped
to HdrR genome
= paternal expression
Compare paternal (= zygotic) expression level of zygotic genes
Control vs α-amanitin at the pre-early gastrula stage
B
Zygotic expression level of zygotic genes
α-amanitin pre-early gastrula
log2 (RPKM + 0.5)
Control pre-early gastrula
log2 (RPKM + 0.5)
C
Cell collection
Mild sonication
ProK & RNaseA
DNA extraction
Compare relative amount
of DNA by qPCR
Relative DNA amount / embryo
NS
Control
α-amanitin
D
Pre-early gastrula
Control
α-amanitin
DAPI
H3K9me3
10 μm
E
H3K9me3 intensity
(nuclei / background)
NS
Control
α-amanitin
F
Rep1
Rep2
Rep3
Control
α-ama nitin
Ctrl
1.4x 1.0x 0.6x 1.0x 1.0x 1.0x 1.0x 1.0x
MK (kDa)
50
30
15
Anti-GAPDH
38
25
Anti-H3K9me3
G
GAPDH
H3K9me3
Relative intensity
Dilution
Control rep1
α-amanitin rep1
Control rep2
α-amanitin rep2
Control rep3
α-amanitin rep3
H
DAPI
Control
α-amanitin
5 μm
I
Contrast of DNA
***
Control
α-amanitin

 © The Author(s)

Figure EV2. Supportive data for Fig. 2.

(**A**) Procedure of analyzing zygotic expression level in α-amanitin-injected embryos using previous dataset (Nakamura et al, 2021). The number of RNA-seq biological replicate is $n = 2$ and 1 for normal embryos and hybrid embryos, respectively. See Methods for the detail. (**B**) Scatterplot indicates that α-amanitin injection impaired zygotic expression at the pre-early gastrula stage in medaka. Previous dataset (Nakamura et al, 2021) was analyzed as shown in (**A**) and Methods. (**C**) Procedure of quantification of relative amount of DNA per embryo (left) and the results (right) at the late blastula stage. Two-sided unpaired Student's t-test. Error bars indicate the mean ± s.d. $n = 4$ biological replicates. (**D**) Immunofluorescence staining of H3K9me3 in α-amanitin-injected medaka embryos at the pre-early gastrula stage. (**E**) Quantification of (**D**). Each dot indicates the average intensity of ~50 cells in a single broad field slice image of single embryo. Two-sided Welch's t-test. Bars indicate the means. $n = 18$ and 19 embryos for the control and α-amanitin, respectively. Data were pooled from two independent experiments. (**F**) Uncropped results of quantitative western blot at the late blastula stage using anti-GAPDH and anti-H3K9me3 antibodies. Magenta arrows indicate the specific bands. (**G**) Quantification of western blot signal intensities in Figs. 1F and EV2F. Scatter plots show that all signal intensities of western blots were within the linear range. (**H**) DAPI staining of control or α-amanitin-injected embryos at the late blastula stage. (**I**) Quantification of (**H**). Each dot indicates the DNA contrast of a single nucleus. Ten embryos were analyzed. Two-sided unpaired Student's t-test. Bars indicate the means. $n = 53$ and 59 nuclei for the late morula and late blastula, respectively. Data were pooled from two independent experiments. ***$p < 0.001$, NS: not significant.

© The Author(s)

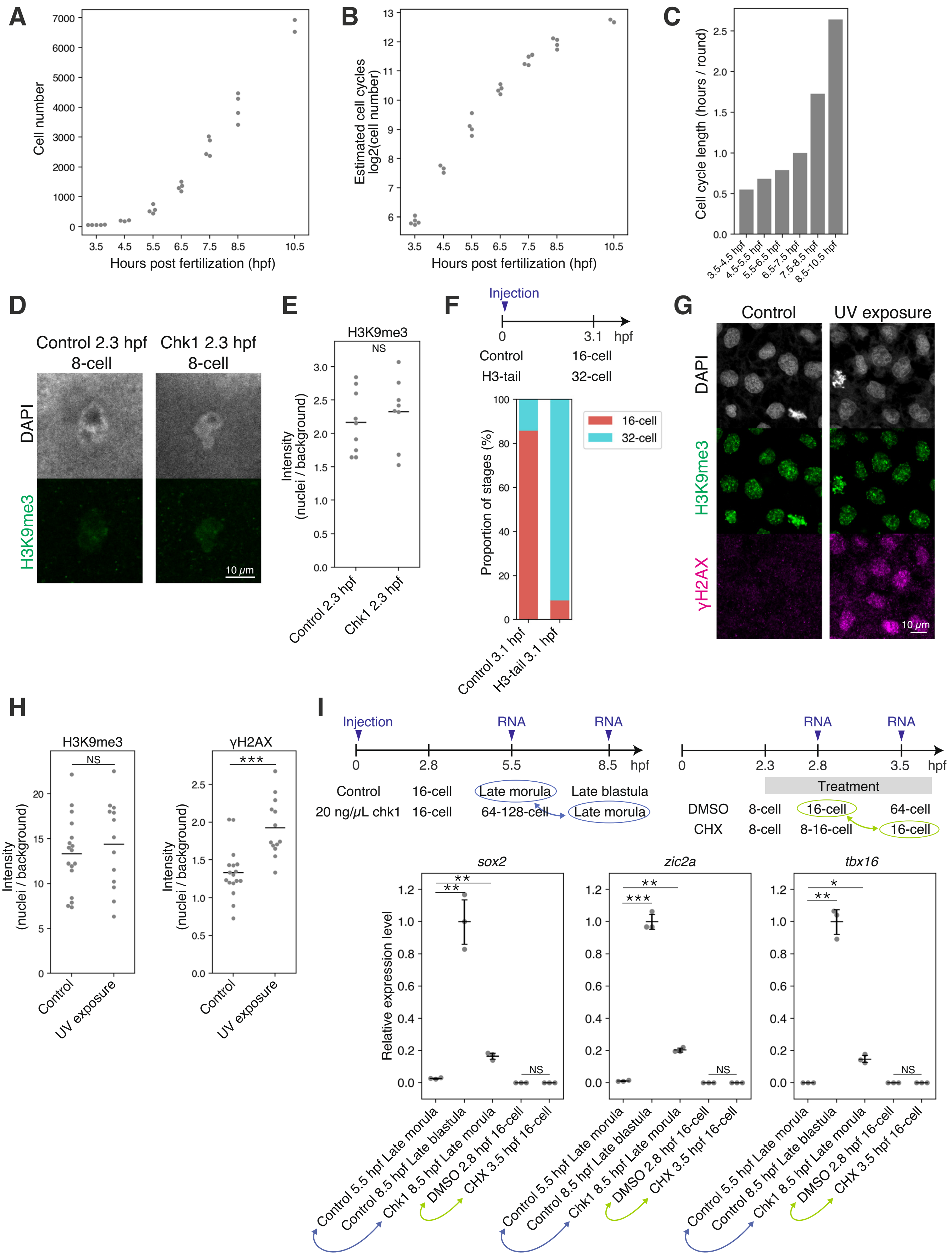
A
Cell number
Hours post fertilization (hpf)
B
Estimated cell cycles
log2(cell number)
Hours post fertilization (hpf)
C
Cell cycle length (hours / round)
3.5-4.5 hpf
4.5-5.5 hpf
5.5-6.5 hpf
6.5-7.5 hpf
7.5-8.5 hpf
8.5-10.5 hpf
D
Control 2.3 hpf
8-cell
Chk1 2.3 hpf
8-cell
DAPI
H3K9me3
10 μm
E
H3K9me3
NS
Intensity
(nuclei / background)
Control 2.3 hpf
Chk1 2.3 hpf
F
Injection
0
3.1 hpf
Control
16-cell
H3-tail
32-cell
Proportion of stages (%)
16-cell
32-cell
Control 3.1 hpf
H3-tail 3.1 hpf
G
Control
UV exposure
DAPI
H3K9me3
γH2AX
10 μm
H
H3K9me3
NS
γH2AX
***
Intensity
(nuclei / background)
Control
UV exposure
I
Injection
RNA
RNA
0
2.8
5.5
8.5
hpf
Control
16-cell
Late morula
Late blastula
20 ng/μL chk1
16-cell
64-128-cell
Late morula
RNA
RNA
0
2.3
2.8
3.5
hpf
Treatment
DMSO
8-cell
16-cell
64-cell
CHX
8-cell
8-16-cell
16-cell
sox2
zic2a
tbx16
Relative expression level
NS
Control 5.5 hpf Late morula
Control 8.5 hpf Late blastula
Chk1 8.5 hpf Late morula
DMSO 2.8 hpf 16-cell
CHX 3.5 hpf 16-cell

 © The Author(s)

Figure EV3. Supportive data for Fig. 3.

(**A**) Number of cells per embryo at 3.5–10.5 hpf counted by Imaris software using DAPI staining data. $n = 5, 3, 4, 4, 4, 4, 2$ embryos for each stage. (**B**) Number of post fertilization cell-cycles estimated by total number of cells per embryo in (**A**). $n = 5, 3, 4, 4, 4, 4, 2$ embryos for each stage. (**C**) Cell cycle length estimated by cell cycle number and time line in (**B**). (**D**) Immunofluorescence staining of H3K9me3 in the chk1 injection experiment at the 8-cell stage (2.3 hpf). (**E**) Quantification of (**D**). Each dot indicates the average of single cells in a single broad field slice image of single embryo. Two-sided unpaired Student's t-test was performed. Bars indicate the means. $n = 9$ and 8 embryos for the control 2.3 hpf and chk1 2.3 hpf, respectively. Data were pooled from two independent experiments. (**F**) Schematic summarizing H3-tail injection (top) and proportion of stages of H3-tail-injected embryos in the cleavage stages (bottom). (**G**) Immunofluorescence staining of H3K9me3 and γH2AX in control and UV-treated embryos at the late blastula stage. (**H**) Quantification of (**G**). Each dot indicates the average of ~100 cells in a single broad field slice image of single embryo. Two-sided unpaired Student's t-test. Bars indicate the means. $n = 17$ and 13 embryos for the control and UV exposure, respectively. Data were pooled from two independent experiments. (**I**) Schematics summarizing RNA sampling (top) and RT-qPCR of zygotic genes (bottom). Expression level was first normalized by that of *actb* in each sample and subsequently normalized by the average expression level in control 8.5 hpf. Stages highlighted in blue or green were compared. Two-sided Welch's t-test. Error bars indicate the mean ± s.d. $n = 3$ biological replicates. *$p < 0.05$, **$p < 0.01$, ***$p < 0.001$, NS: not significant.

© The Author(s)

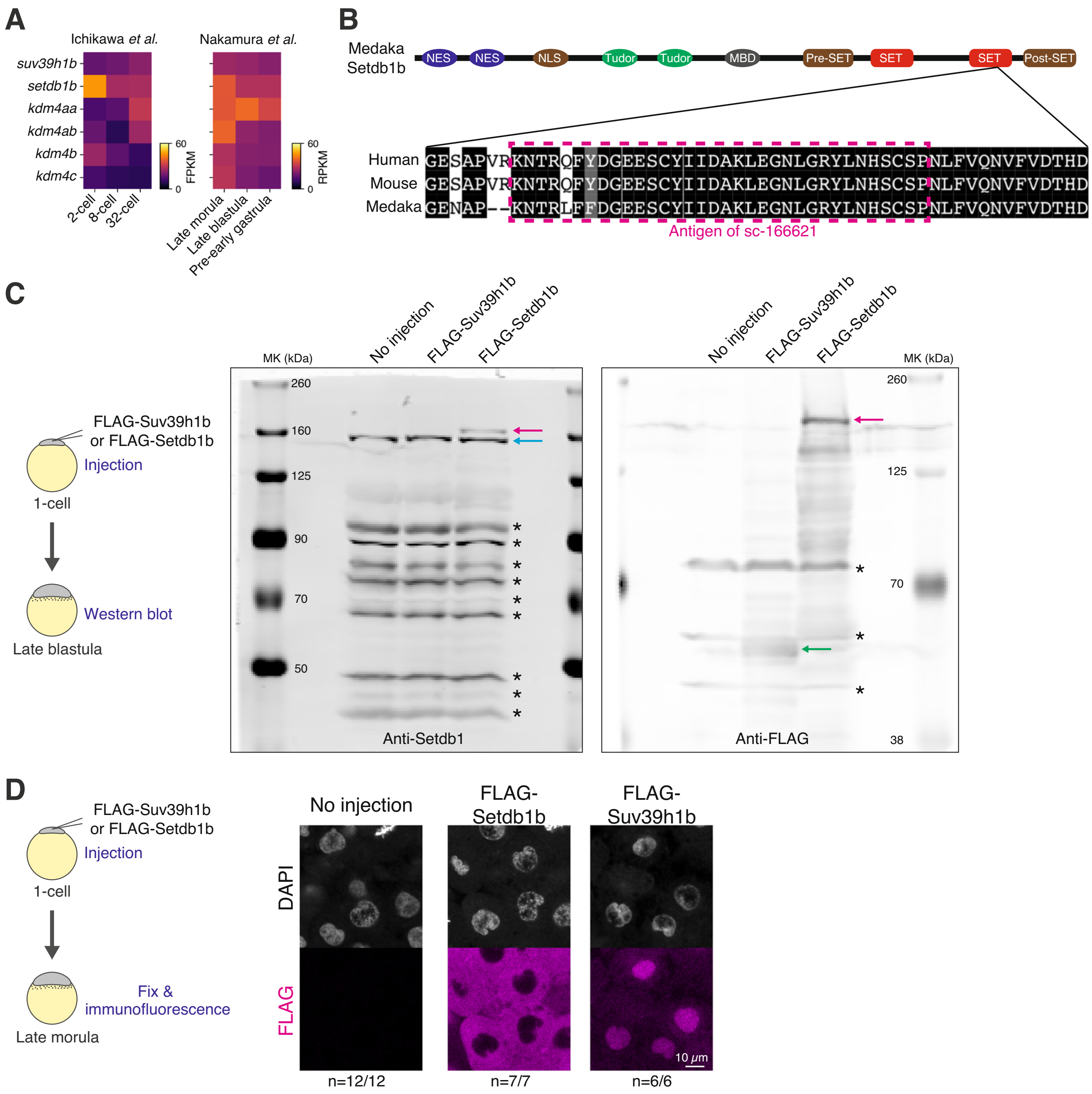

Figure EV4. Supportive data for Fig. 4.

(**A**) Expression level of H3K9me3 methyltransferases and demethylases during early development. Data was obtained from previous RNA-seq data (Nakamura et al, 2021; Ichikawa et al, 2017). (**B**) Domains of Setdb1 and amino acid sequence in the SET domain. The antigen sequence of the anti-Setdb1 antibody (sc-166621) is highlighted in magenta. (**C**) Schematic of the experiments to validate the specificity of the anti-Setdb1 antibody (sc-166621) (left) and the results of western blot at the late blastula stage (right). Blue, magenta, and green arrows indicate endogenous Setdb1b, exogenously expressed FLAG-Setdb1b, and exogenously expressed FLAG-Suv39h1b, respectively. Asterisks (*) indicate non-specific bands. (**D**) Schematic of the experiments to validate the localization of exogenously expressed FLAG-Suv39h1 and FLAG-Setdb1 (left) and immunofluorescence staining against anti-FLAG at the late morula stage (right). Consistent with the Fig. 4, exogenously overexpressed FLAG-Setdb1 localized to cytoplasm, while FLAG-Suv39h1 mainly accumulated in nuclei. The number of embryos with the representative pattern is indicated at the bottom.

 © The Author(s)

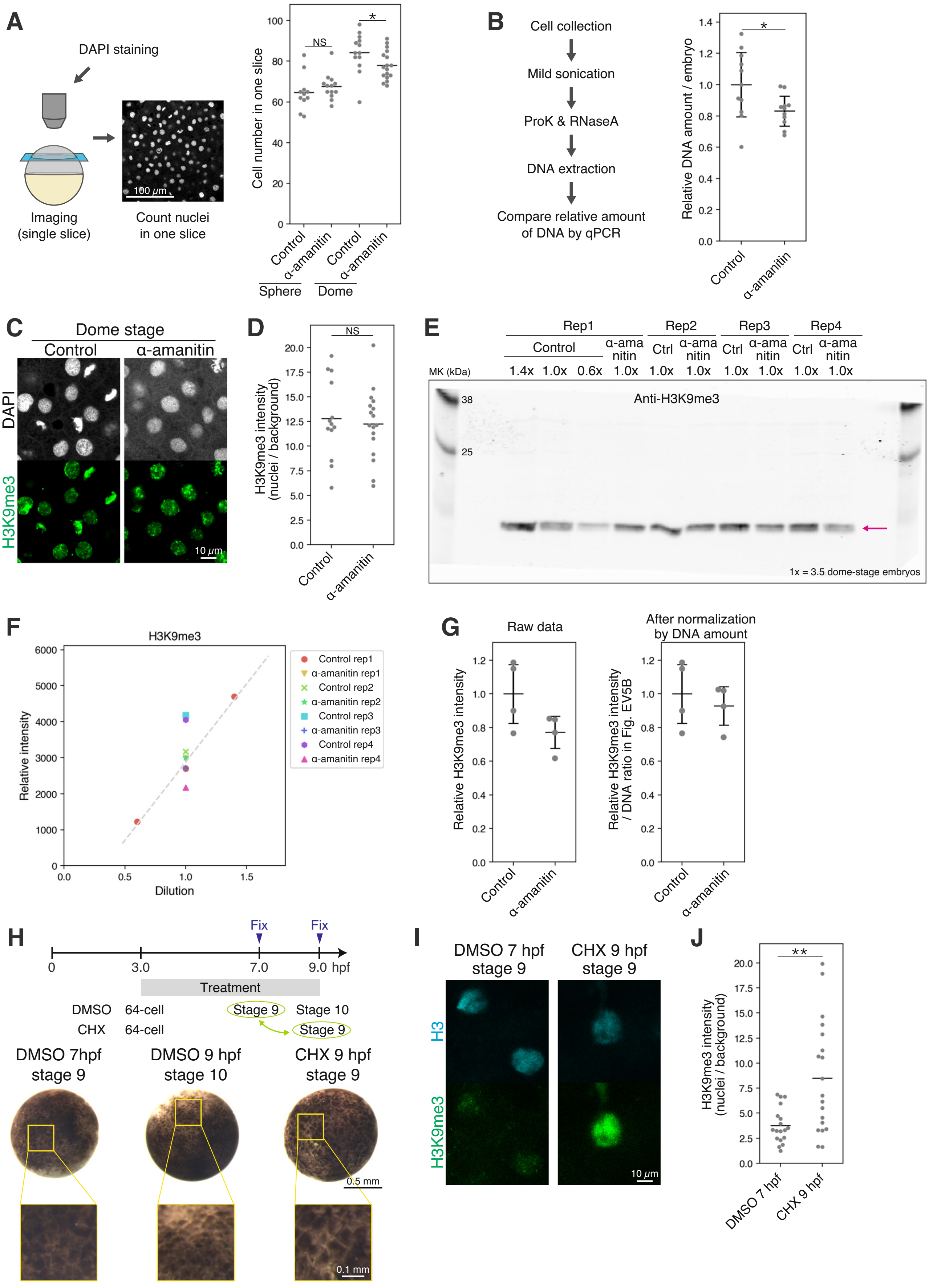
A
DAPI staining
Imaging (single slice)
Count nuclei in one slice
100 μm
Cell number in one slice
NS
*
Control
α-amanitin
Sphere
Dome
B
Cell collection
Mild sonication
ProK & RNaseA
DNA extraction
Compare relative amount of DNA by qPCR
Relative DNA amount / embryo
C
Dome stage
Control
α-amanitin
DAPI
H3K9me3
10 μm
D
H3K9me3 intensity (nuclei / background)
E
Rep1
Rep2
Rep3
Rep4
Ctrl
α-amanitin
MK (kDa)
Anti-H3K9me3
1x = 3.5 dome-stage embryos
F
H3K9me3
Relative intensity
Dilution
Control rep1
α-amanitin rep1
G
Raw data
After normalization by DNA amount
Relative H3K9me3 intensity
Relative H3K9me3 intensity / DNA ratio in Fig. EV5B
H
Fix
Treatment
DMSO
CHX
64-cell
Stage 9
Stage 10
DMSO 7hpf stage 9
DMSO 9 hpf stage 10
CHX 9 hpf stage 9
0.5 mm
0.1 mm
I
DMSO 7 hpf stage 9
CHX 9 hpf stage 9
H3
J
**
DMSO 7 hpf
CHX 9 hpf

Figure EV5. Supportive data for Figs. 5 and 6.

(**A**) Schematic of counting nuclei in an embryo using a single slice (left) and the cell number in α-amanitin-injected zebrafish embryos at the sphere or dome stage (right). Two-sided unpaired Student's t-test. Bars indicate the means. $n = 11$, 13, 13, and 17 embryos for the sphere control, sphere α-amanitin, dome control, and dome α-amanitin, respectively. Data were pooled from two independent experiments. (**B**) Procedure of quantification of the relative amount of DNA per embryo (left) and the results (right) in α-amanitin-injected zebrafish embryos at the dome stage. Two-sided Welch's t-test. Error bars indicate the mean ± s.d. $n = 11$ biological replicates. (**C**) Immunofluorescence staining of H3K9me3 in α-amanitin injection experiment at the dome stage. (**D**) Quantification of (**C**). Each dot indicates the average of ~80 cells in a single broad field slice image of single embryo. Two-sided unpaired Student's t-test. Bars indicate the means. $n = 13$ and 17 embryos for the control and α-amanitin, respectively. Data were pooled from two independent experiments. (**E**) Uncropped results of quantitative western blot at the dome stage using anti-H3K9me3 antibody. The Magenta arrow indicates the specific bands. The same number of dome-stage embryos (1× = ~3.5 embryos/lane) were loaded into each lane to compare total H3K9me3 levels per embryo. (**F**) Quantification of western blot signal intensities in (**E**). Scatter plots show that all western blot signal intensities were within the linear range. (**G**) Quantification of (**E**). On the right, data after normalization by the DNA ratio measured in Fig EV5B. Error bars indicate the mean ± s.d. $n = 4$ biological replicates. (**H**) Schematic summarizing the CHX treatment (top) and animal view of CHX-treated *X. laevis* embryos (bottom). Stages highlighted in green were compared in (**I**) and (**J**). To compare developmental stages, cells at the animal poles are magnified (yellow squares). (**I**) Immunofluorescence staining of H3 and H3K9me3 in CHX treatment at the stage 9. (**J**) Quantification of (**I**). Each dot indicates the average of ~5–10 cells in a single broad field slice image of single embryo. Two-sided Welch's t-test. Bars indicate the means. $n = 18$ and 19 embryos for the DMSO 7 hpf and CHX 9 hpf, respectively. Data were pooled from two independent experiments. *$p < 0.05$, **$p < 0.01$, NS: not significant.

 © The Author(s)
